# Structure‐based design of antibodies targeting the EBNA1 DNA‐binding domain to block Epstein–Barr virus latent infection and tumor growth

**DOI:** 10.1002/mco2.739

**Published:** 2024-10-10

**Authors:** Yongyue Han, Fang Wu, Ying Zhang, Jun Liu, Yuzhe Wu, Yuecheng Wang, Xiwen Jiang, Xin Chen, Wei Xu

**Affiliations:** ^1^ Guangdong Provincial Key Laboratory of New Drug Screening & NMPA Key Laboratory for Research and Evaluation of Drug Metabolism & Guangdong‐Hong Kong‐Macao Joint Laboratory for New Drug Screening School of Pharmaceutical Sciences Southern Medical University Guangzhou China; ^2^ Affiliated Foshan Maternity & Child Healthcare Hospital Southern Medical University Foshan China; ^3^ School of Life Sciences and Biopharmaceuticals Guangdong Pharmaceutical University Guangzhou China; ^4^ Department of Pulmonary and Critical Care Medicine Zhujiang Hospital Southern Medical University Guangzhou China; ^5^ Key Laboratory of Infectious Diseases Research in South China Ministry of Education Southern Medical University Guangzhou China

**Keywords:** Epstein–Barr virus latent infection, Epstein–Barr virus nuclear antigen 1, monoclonal antibody, tumor

## Abstract

The Epstein–Barr virus (EBV) nuclear antigen 1 (EBNA1) is critically involved in maintaining episomes during latent infection and promoting tumorigenesis. The development of an epitope‐specific monoclonal antibody (mAb) for EBNA1 holds great promise due to its high affinity and specificity, offering a new and innovative approach for the treatment of EBV‐related diseases. In this proof‐of‐concept study, we employed a structure‐based design strategy to create three unique immunogens specifically targeting the DNA binding state of the EBNA1 DBD. By immunizing mice, we successfully generated a mAb, named 5E2‐12, which selectively targets the DNA binding interface of EBNA1. The 5E2‐12 mAb effectively disrupts the interaction between EBNA1 and DNA binding, resulting in reduced proliferation of EBV‐positive cells and inhibition of xenograft tumor growth in both cellular assays and mouse tumor models. These findings open up new avenues for the development of innovative biological macromolecular drugs that specifically target EBNA1 and provide potential for clinical therapy options for early‐stage EBV‐positive tumors. The epitope‐specific mAb approach demonstrates novelty and innovation in tackling EBV‐related diseases and may have broad implications for precision medicine strategies in the field of viral‐associated cancers.

## Graphical abstract

1. Engineering of epitope‐directed antibodies against EBNA1 can block DNA binding in vitro and in vivo.
2. Site 1 on EBNA1 DNA‐binding domain is crucial for binding DNA and maintaining EBV latent infections.
3. Monoclonal antibody 5E2‐12 can inhibit EBV positive tumors growth by targeting EBNA1 site 1.

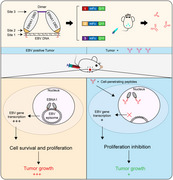

## 1 INTRODUCTION

Epstein–Barr virus (EBV) is a ubiquitous human DNA virus that typically causes asymptomatic infection in at least 95% of adults.^1^, ^2^ In adolescents, the initial EBV infection can lead to infectious mononucleosis, a self‐limited disease.^3^ However, as a member of the human herpesvirus 4 family, EBV has the ability to infect host cells and establish both lytic and latent infections.^4^ During latency, the EBV genome remains stably present in the host cell nucleus in the form of episomes.^5^ While the human immune system can effectively control EBV latent infection, the prolonged presence of viral episomes can increase the risk of developing certain malignancies.^6^ Extensive research has shown that EBV latent infection serves as a trigger for various cancers originating from lymphocytes and epithelial cells.^7^ Currently, clinical medications such as acyclovir, which are DNA polymerase inhibitors, can only effectively inhibit viral lytic infections. Therefore, there is a need to develop novel therapeutic strategies to target latent EBV infection.

Epstein–Barr nuclear antigen 1 (EBNA1) is a viral protein with a high affinity for DNA binding. Current research has demonstrated that the expression of EBNA1 is associated with various human diseases, including tumors and autoimmune diseases such as multiple sclerosis.^8^, ^9^ EBNA1 plays a crucial role in maintaining the EBV genome as episomes within the host nucleus during EBV latency. By interacting with viral episomes, EBNA1 initiates DNA replication and regulates viral gene expression, which leads to increased survival and immortalization of primary B lymphocytes.^10^ EBNA1 expression is detected in all EBV‐associated malignancies, including Burkitt's lymphoma, nasopharyngeal carcinoma (NPC), Hodgkin's lymphoma, and gastric carcinoma.^11^, ^12^ Recent studies have highlighted the close association between EBNA1 and tumor initiation, primarily due to its nonfunctional interaction with human chromosome 11q23.^13^ These functions of EBNA1 highlight its significance as a potential therapeutic target for EBV‐associated malignancies.

The structure of the DNA‐binding domain (DBD) of EBNA1 has been successfully determined using X‐ray crystallography, providing valuable insights into its functional role.^14^, ^15^ This structural information provides a foundation for the rational design and development of targeted therapeutics that can effectively inhibit the function of the EBNA1 DBD. Several small‐molecule compounds targeting specific sites on the EBNA1 DBD have been identified through high throughput screening based on the available crystal structure data.^16^ However, comprehensive evaluation of their in vitro and in vivo toxicity and safety profiles is still needed. Monoclonal antibodies (mAbs) are highly specific biomolecules that can target protein epitopes with potentially minimal toxicity both in vitro and in vivo. Over 175 mAb therapeutics have been approved or are currently under regulatory review.^17^, ^18^ Previous studies have demonstrated the efficacy of mAbs targeting oncogenic Ras mutants in exerting antitumor effects.^19^ This highlights the feasibility of developing antibody‐based therapeutics that specifically target intracellular EBNA1 for the treatment of EBV‐associated malignancies.

In this study, we designed immunogens specifically engineered to elicit targeted antigenic responses for the generation of epitope‐directed antibodies. Through screening individual hybridomas clones, we successfully identified a mAb designated as 5E2‐12. Notably, when combined with a cell‐penetrating peptide (CPP), the 5E2‐12 mAb exhibited significant antitumor activity against EBV‐positive NPC and Burkitt's lymphoma in both in vitro and in vivo models. Importantly, the 5E2‐12 mAb did not demonstrated any effect on EBV‐negative tumors models. These findings highlight the potential clinical applications of 5E2‐12 as a promising therapeutic option for the treatment of EBV‐related tumors.

## 2 RESULTS

### 2.1 Engineering of epitope‐directed immunogens on EBNA1 DBD

**FIGURE 1:**
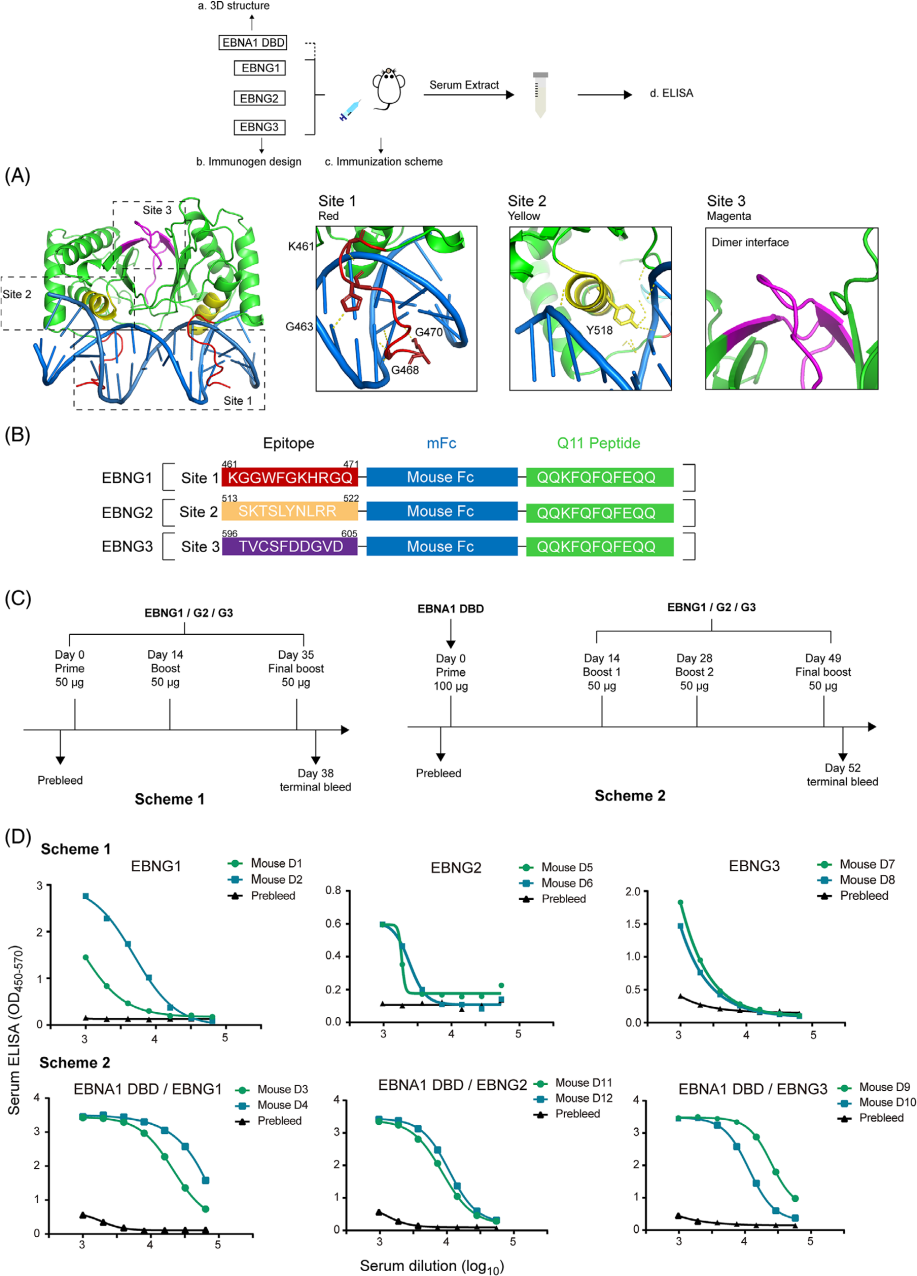
Design of Structure‐guided epitope‐directed immunogens for eliciting EBNA1 DBD binding responses. (A) X‐ray crystal structures showing the three epitopes (site 1 in red, site 2 in yellow, site 3 in magenta) within the EBNA1 DBD (green ribbon), which are involved in blocking DNA (blue ribbon) binding. The enlarged image on the right highlights the contact sites. (B) Schematic representation of the fusion peptide‐based immunogens designed for immunizing BALB/c mice. (C) Two different immunization schemes employed for BALB/c mice, with epitope‐directed immunogens on the left and combined immunogens on the right. (D) Evaluation of EBNA1 DBD binding responses in mice serum after immunization, assessed by ELISA on day 38 (above) and day 52 (below).

To develop potential mAb for the treatment of EBNA1‐related diseases, we employed a rational design approach based on the three‐dimensional structure of EBNA1 bound to its DNA sequence (PDB 1B3T) and previous studies on small‐molecule inhibitors targeting the EBNA1 DBD. Through our structural analysis, we identified three specific sites on EBNA1 DBD that were promising candidates for targeted epitope‐directed antibody generation: site 1 and site 2 located at the DNA‐binding interface, and site 3 positioned adjacent to a dimer interface (Figure 1A).

To enhance the immunogenicity of these targeted epitopes, we generated peptide–carrier protein conjugates using a mouse Fc and Q11, a self‐assembling peptide known to improve immunogenicity while minimizing inflammation through the formation of nanofibers and hydrogels (Figure 1B).^20^ This approach aimed to generate a robust immune response against the targeted EBNA1 epitopes.

To increase the likelihood of successfully generating mAbs, we employed two distinct immunization schemes. In Scheme 1, we used peptide derived from the identified epitopes as the immunogens, while in Scheme 2, we first immunized mice with the EBNA1 DBD protein, followed by subsequent immunizations with the epitope‐derived peptides (Figure 1C). These schemes followed the established protocol outlined by Xu et al.,^21^ optimizing the immunization process to maximize the production of desired antibodies.

To evaluate the ability of the epitope‐based immunogens (named EBNG1, EBNG2, and EBNG3) to elicit binding responses against EBNA1 DBD, we performed enzyme‐linked immunosorbent assays (ELISA). We assessed the presence of strong binding responses against EBNA1 DBD in the serum samples collected on day 38 (Scheme 1) and day 52 (Scheme 2) following immunization with the EBNG antigens.

Our results revealed robust and specific binding responses against EBNA1 DBD in the serum samples from both immunization schemes. Notably, the immunogen EBNG1 demonstrated particularly strong binding activity in the serum collected on day 38 in Scheme 1, surpassing the effectiveness of EBNG2 (Figure 1D).

### 2.2 5E2‐12 mAb target the EBNG1 epitope with high affinity

The presence of high levels of binding responses against EBNA1 DBD indicates the secretion of high‐affinity IgG antibodies that specifically target residues 461−471 of EBNA1 DBD. To isolate and extract the antibodies elicited by the EBNG1‐containing immunogens, we selected B cell hybridomas capable of binding to EBNA1 DBD using B cells obtained from the mouse D2, which had been immunized with EBNG1 alone. Through a screening process, we identified hybridomas from clone number 5E2 and subsequently cultured subclones (5E2‐3, 5, 9, 12).

**FIGURE 2:**
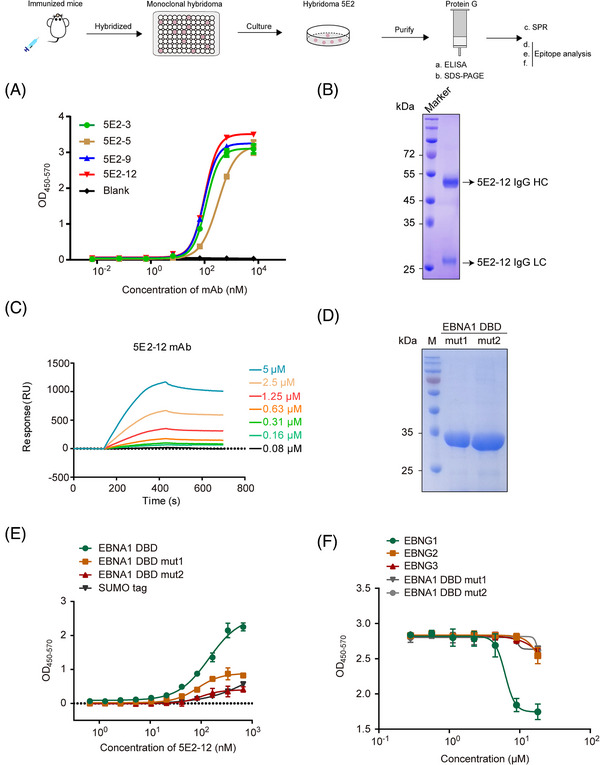
Affinity and epitope validation of isolated antibodies. (A) ELISA was employed to determine the affinity of the isolated antibodies: 5E2‐3 (EC_50_: 116.3 nM), 5E2‐5 (EC_50_: 320.6 nM), 5E2‐9 (EC_50_: 96.18 nM), and 5E2‐12 (EC_50_: 101.9 nM). (B) Coomassie Brilliant Blue staining in SDS‐PAGE was utilized to visualize the extraction of the 5E2‐12 mAb. (C) Surface plasmon resonance analysis was conducted to determine the affinity of the 5E2‐12 mAb. (D) Coomassie Brilliant Blue staining in SDS‐PAGE was utilized to visualize the expression and purification of the mutant 1 (mut1) and mutant 2 (mut2) from EBNA1 DBD. (E) ELISA assay was employed to determine the affinity of the 5E2‐12 mAb to EBNA1 DBD, EBNA1 DBD mut1 and EBNA1 DBD mut2. SUMO tag as a negative control. (F) Competitive ELISA was employed to detect the binding of the 5E2‐12 mAb with EBNA1 DBD, EBNG1, EBNG2, and EBNG3. EBNA1 DBD mut1 and mut 2 as a negative control.

To determine the affinity of these subclones for EBNA1 DBD, we performed ELISA. Among the subclones, clone 5E2‐12 exhibited an EC_50_ value of approximately 100 nM (Figure 2A), indicating a binding affinity for EBNA1 DBD.

The 5E2‐12 mAb, secreted by the 5E2‐12 hybridoma cell line, was further purified to evaluate its binding characteristics (Figure 2B). We confirmed that the 5E2‐12 mAb exhibited a direct binding to EBNA1 DBD, with a *K*_D_ value of 196 nM (Figure 2C). This finding underscores the interaction between the 5E2‐12 mAb and EBNA1 DBD, further supporting its potential as a therapeutic agent.

Additionally, we cloned and purified two additional constructs of EBNA1 DBD protein, where the 461−471 residues were either mutated (mutant 1) or removed (mutant 2) (Figure 2D). We found that the binding effect with 5E2‐12 mAb was eliminated in mutant 1 (mut1) and mutant 2 (mut2) of EBNA1 DBD (Figure 2E). We then conducted competitive binding assays to assess the specificity of the 5E2‐12 mAb. The results demonstrated that the blockade of EBNG1 immunogen partially eliminated the binding of 5E2‐12 mAb to EBNA1 DBD, while the EBNG2 and EBNG3 immunogen could not (Figure 2F). This confirms the specific targeting of residues 461−471 of EBNA1 DBD by the 5E2‐12 mAb, validating its epitope‐specificity.

Unfortunately, in this particular part of the study, no valuable mAbs were identified from Scheme 2. Despite this outcome, the successful isolation and characterization of the 5E2‐12 mAb from Scheme 1 demonstrate the efficacy of our immunization approach and the potential of the EBNG1 immunogen in generating highly specific and high‐affinity antibodies against EBNA1 DBD.

### 2.3 5E2‐12 mAb blocks DNA binding and inhibits proliferation of EBV^+^ tumor cells in vitro

**FIGURE 3:**
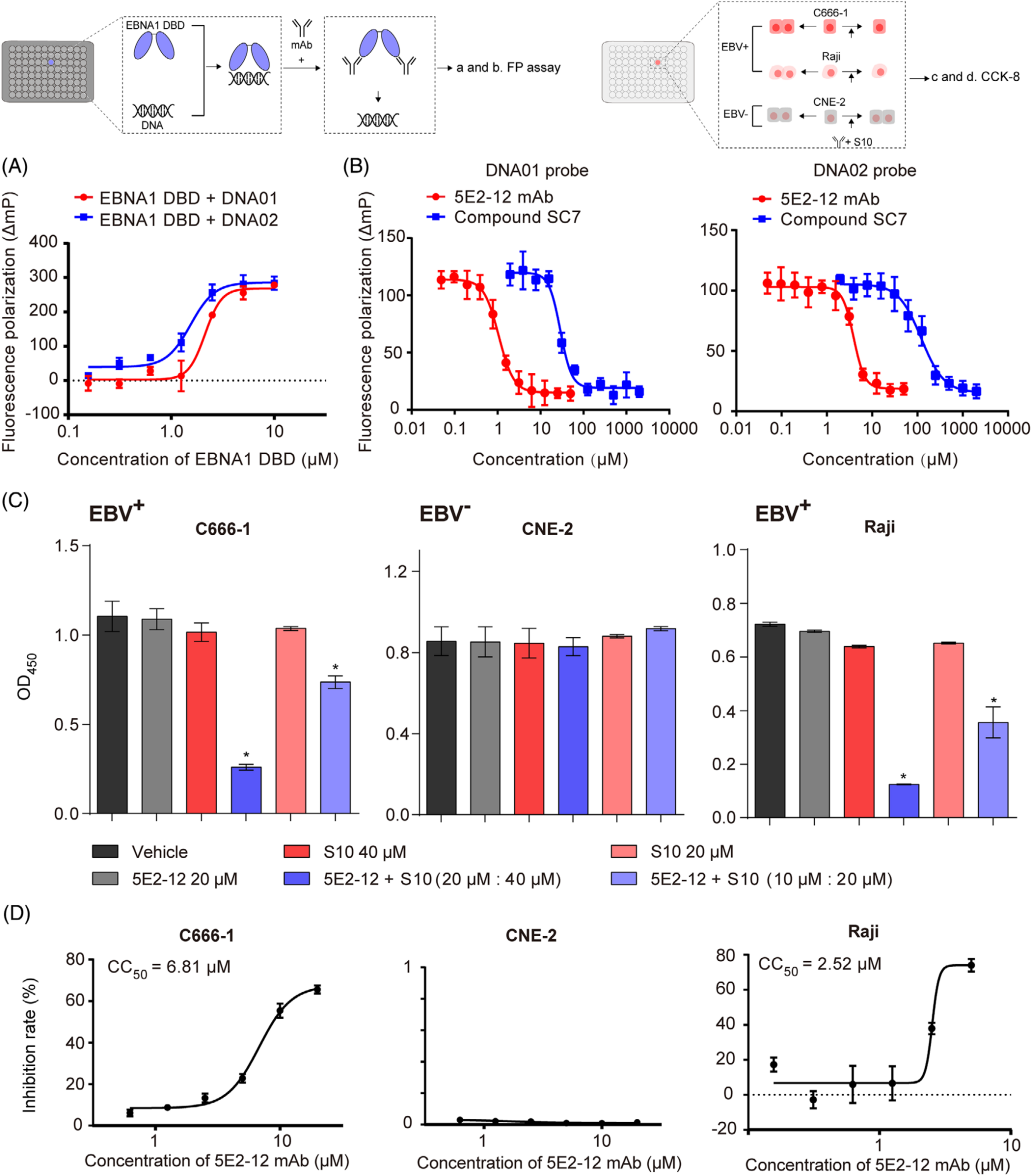
Effect of 5E2‐12 mAb on blocking DNA binding and inhibiting proliferation of EBV^+^ tumor cells. (A) FP was employed to detect the binding of EBNA1 DBD with two DNA probes. (B) The capacity of 5E2‐12 mAb or compound SC7 to disrupt the binding observed in (A) was assessed. (C) CCK‐8 assay was utilized to evaluate the inhibitory effect of 5E2‐12 mAb on EBV^+^ and EBV^−^ tumor cells. (D) CCK‐8 assay was employed to draw a dose–response curve of 5E2‐12 mAb on EBV^+^ and EBV^−^ tumor cells. *p < 0.05 compared with the vehicle group.

The DNA binding capacity of the recombinantly expressed EBNA1 DBD in *Escherichia coli* was investigated to assess its interaction with specific DNA sequences. We tested the affinity of EBNA1 DBD for an 18‐base‐pair (bp) DNA probe derived from EBV episomes (DNA01) and an 18‐bp DNA probe from human chromosome 11q23 (DNA02). The results revealed high‐affinity interactions between EBNA1 DBD and both DNA probes, with EC_50_ values of 2.13 µM for DNA01 and 1.54 µM for DNA02 (Figure 3A). This confirms the ability of EBNA1 DBD to bind to these specific DNA sequences.

To explore the potential of targeting EBNA1 DBD site 1 to block DNA binding, we investigated the disruption of the interactions between EBNA1 DBD and DNA probes. We evaluated the effect of the 5E2‐12 mAb, which specifically targets residues 461−471 of EBNA1 DBD, and compound SC7, a known control that blocks DNA binding in EBNA1.^22^ Both the 5E2‐12 mAb (IC_50_: 1.07 µM on DNA01, approx. 3.92 µM on DNA02) and compound SC7 (IC_50_: 28.95 µM on DNA01, approx. 121.60 µM on DNA02) effectively abrogated the interaction between EBNA1 DBD and the DNA probes, demonstrating their ability to inhibit DNA binding (Figure 3B).

Having confirmed the inhibitory effect of the 5E2‐12 mAb on EBNA1 DBD–DNA interactions, we proceeded to assess its impact on cell proliferation. To do this, we combined the 5E2‐12 mAb with S10, a CPP that can efficiently transfer proteins into cells.^23^ We examined the proliferation of EBV‐positive tumor cells and EBV‐negative tumor cells in the presence of the 5E2‐12 mAb and S10 combined with different doses. The results demonstrated that the combination of the 5E2‐12 mAb and S10 significantly inhibited the proliferation of EBV‐positive tumor cells (Figure 3C). Then, we kept the concentration of S10 at 20 µM to explore the dose–response curve of inhibiting tumor cell proliferation. Importantly, this inhibitory effect was specific to EBV‐positive tumor cells, as the 5E2‐12 mAb had no significant impact on the proliferation of EBV‐negative tumor cells (Figure 3D). To ensure the validity of subsequent mAb activity assessments, the CNE‐2 cell line was excluded due to potential contamination with HeLa cells, another EBV‐negative tumor cell line.^24^, ^25^ To maintain experimental consistency, an alternative EBV‐negative NPC cell line, HK1, was selected, and the results obtained using this cell line were consistent with those obtained previously (Figure S2). These findings highlight the potential of the 5E2‐12 mAb in selectively targeting and impeding the growth of EBV‐positive tumor cells.

Furthermore, the combination of the 5E2‐12 mAb with the CPP S10 enhances its potential as a therapeutic strategy. The CPP facilitates the delivery of the 5E2‐12 mAb into cells, ensuring its efficient and targeted action against EBV‐positive tumor cells while minimizing effects on healthy cells.

### 2.4 5E2‐12 mAb reduces DNA synthesis and EBV gene expression in vitro

In line with our hypothesis, the observed decrease in proliferation of EBV‐positive cells induced by the 5E2‐12 mAb can be attributed to a reduction in intracellular DNA synthesis. To investigate this further, we utilized 5‐ethynyl‐2′‐deoxyuridine (EdU), a nucleoside analog that labels replicating DNA within the nucleus. By incorporating EdU into newly synthesized DNA, we can visualize and quantify the extent of DNA synthesis.

**FIGURE 4:**
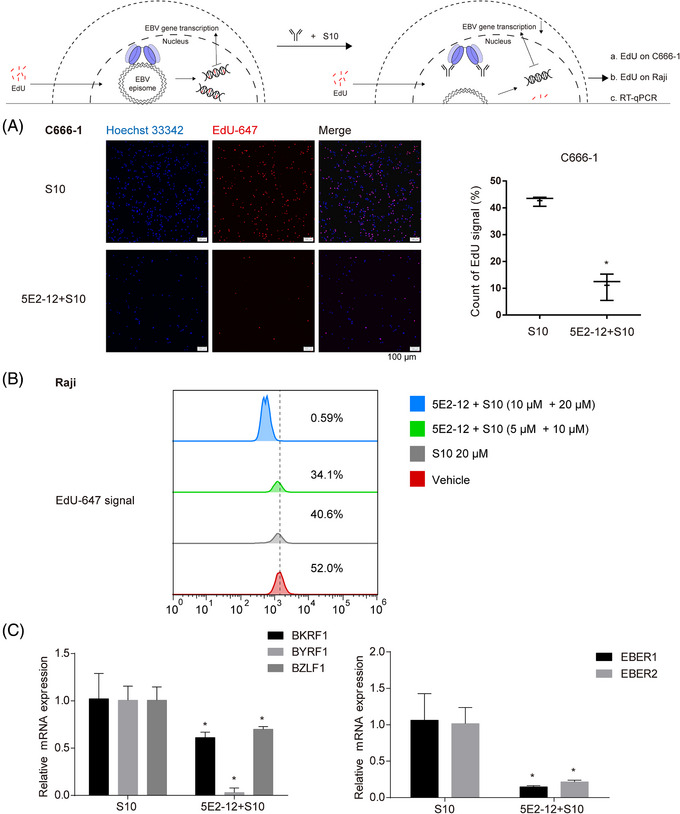
Effect of 5E2‐12 mAb on DNA synthesis and viral gene expression in C666‐1 cells and Raji cells. (A) DNA synthesis was detected using EdU stain and visualized under a fluorescence microscope in C666‐1 cells (scale bar: 100 µm). (B) Flow cytometry analysis was performed to assess DNA synthesis in Raji cells using EdU stain. (C) RT‐qPCR was employed to measure the mRNA expression levels of BKRF1, BYRF1, BZLF1, EBER1, and EBER2 in Raji cells. *p < 0.05 compared with the S10 group.

In our study, we examined the effect of 5E2‐12 mAb intervention on DNA synthesis by labeling replicating DNA with EdU. The total number of C666‐1 cells, an EBV‐positive cell line, was reduced in the 5E2‐12 (20 µM) + S10 (40 µM) treatment group compared with the control group (Figure 4A). This observation suggests that the combination of 5E2‐12 mAb and S10, a CPP, leads to a decrease in cell proliferation.

To further investigate the impact of 5E2‐12 mAb on DNA synthesis, we performed click chemistry with Azide Alexa Fluor 647 to detect EdU‐labeled DNA. We found that the EdU‐647 signal ratio, which represents the level of DNA synthesis, was decreased in the 5E2‐12 + S10 treatment group compared with the S10 group in both C666‐1 cells and Raji cells (Figure 4A,B). This reduction in the EdU‐647 signal indicates a decrease in ongoing DNA replication, supporting the notion that 5E2‐12 mAb intervention hinders DNA synthesis in EBV‐positive cells.

In addition to the inhibition of DNA synthesis, we investigated whether the decrease in DNA replication was accompanied by a reduction in EBV transcripts. We evaluated the expression levels of EBV‐encoded RNA (EBER1 and EBER2), small noncoding RNAs predominantly transcribed during EBV latent or lytic infection. We also assessed the copy number of EBERs as a measure of EBV latent infection.

As anticipated, treatment with 5E2‐12 mAb resulted in a significant decrease in mRNA expression levels of the three targets (EBER1, EBER2, and the additional target) (Figure 4C and Table S1). The enhanced levels of BKRF1 (EBNA1 gene), BYRF1 (EBNA2 gene), and BZLF1 (Zta ZEBRA gene) mRNA, which are EBV‐related genes, reflect the latent infection process in the EBV‐positive tumor cells. Moreover, the copy number of EBERs, indicative of EBV latent infection, was also significantly reduced following 5E2‐12 mAb intervention. These findings suggest that 5E2‐12 mAb not only hampers DNA synthesis but also leads to a decrease in EBV transcript levels, thereby affecting the latent infection of EBV.

Taken together, our results provide evidence for the mechanism underlying the decreased proliferation of EBV‐positive cells induced by 5E2‐12 mAb. The combination of 5E2‐12 mAb and S10 impedes DNA synthesis, as demonstrated by a reduction in the total number of cells and the decreased EdU‐647 signal ratio, indicating a decline in ongoing DNA replication. Furthermore, the decrease in DNA synthesis is accompanied by a reduction in EBV transcript levels and the copy number of EBERs, indicating an impact on the latent infection of EBV.

### 2.5 5E2‐12 mAb targets EBNA1 and interferes with its DNA binding function

**FIGURE 5:**
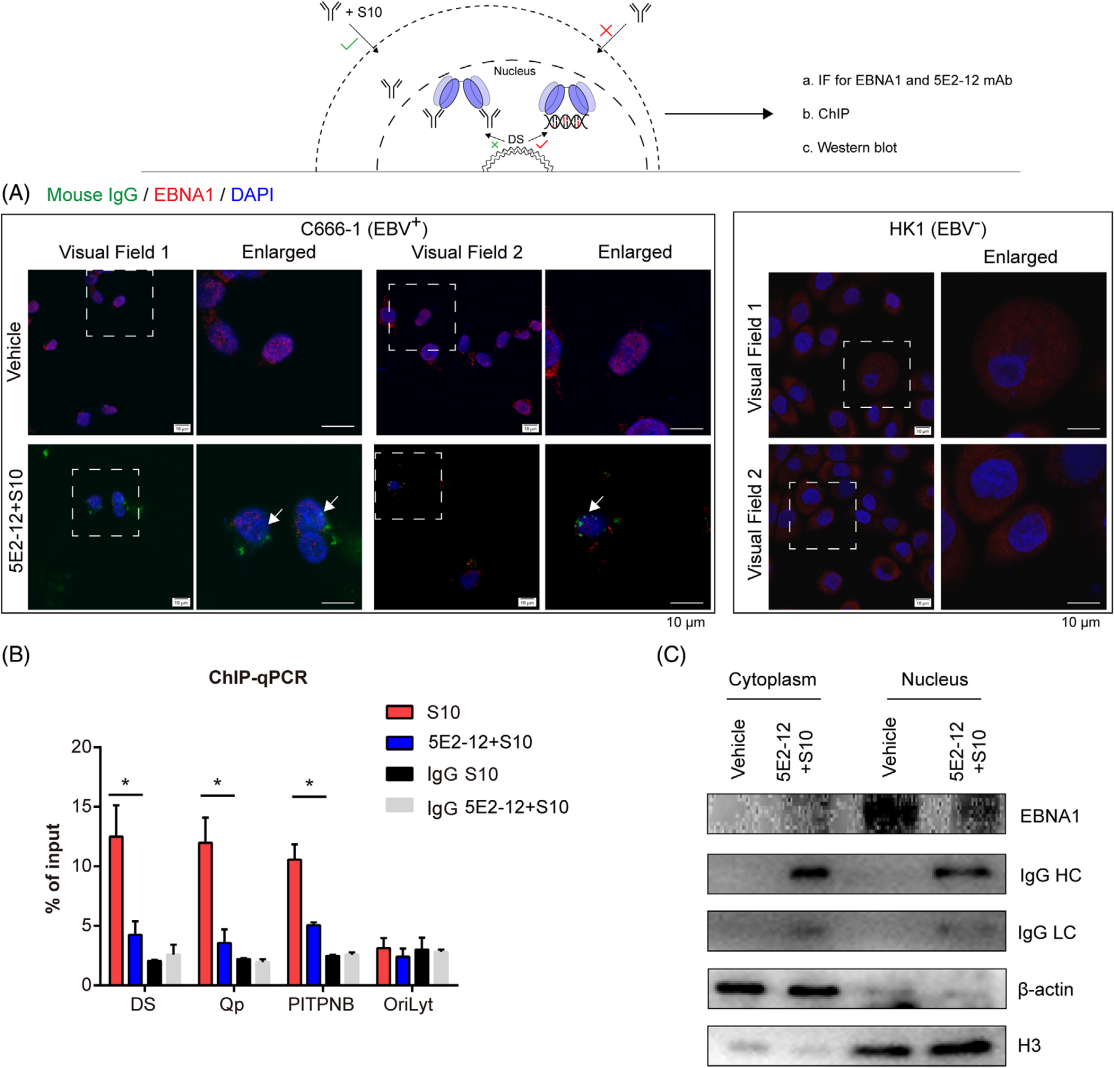
Effect of 5E2‐12 mAb on EBNA1 localization in the nucleus and EBNA1 DNA binding function. (A) IF analysis was used to observe the localization of EBNA1 (red) and 5E2‐12 (green) in C666‐1 cells. Colocalization is indicated by white arrows. Two random visual fields were placed. Scale bar: 10 µm. (B) ChIP assay was performed to examine the binding of EBNA1 to the DS, Qp, PITPNB cellular locus, or negative control EBV OriLyt locus in EBV‐positive Raji lymphoma cells treated with 5 µM 5E2‐12 mAb and 10 µM S10. *p < 0.05 compared with the S10 group. IgG (negative control). (C) Western blot analysis was used to observe the localization of EBNA1 and 5E2‐12 in Raji cells.

To validate the specific targeting of EBNA1 by the 5E2‐12 mAb, we examined the colocalization of EBNA1 and 5E2‐12 in the EBV‐positive NPC cell line C666‐1 using immunofluorescence staining and in the EBV‐positive lymphoma cell line Raji using Western blot analysis (Figure 5A,C). Our results demonstrated colocalization of EBNA1 and 5E2‐12 signals, indicating that the antibody specifically targets EBNA1 within the nucleus. This observation further supports the notion that the 5E2‐12 mAb can directly interact with EBNA1.

To further investigate the impact of 5E2‐12 mAb treatment on EBNA1's DNA‐binding function, we performed a chromatin immunoprecipitation (ChIP) assay. In the EBV‐positive Burkitt's lymphoma cell line Raji, treated with 5 µM 5E2‐12 and 10 µM S10, we assessed EBNA1 binding at various sites, including EBV dyad symmetry (DS) and the EBV Qp, and a cellular binding site at the PITPNB gene (Figure 5B). Our results demonstrated a significant decrease in EBNA1 binding at these genomic loci upon 5E2‐12 mAb treatment, indicating that the antibody interferes with EBNA1's ability to bind to specific DNA sequences.

Collectively, our in‐cell experiments provide compelling evidence for the ability of the 5E2‐12 mAb to interfere with the DNA‐binding function of EBNA1. The ChIP assay results demonstrate a significant decrease in EBNA1 binding at specific DNA sites upon 5E2‐12 mAb treatment, further confirming the antibody's ability to disrupt EBNA1's DNA‐binding capacity. Additionally, the colocalization of EBNA1 and 5E2‐12 mAb signals corroborates the specific targeting of EBNA1 by the antibody.

### 2.6 5E2‐12 mAb inhibits the growth of EBV‐positive tumors in vivo

**FIGURE 6:**
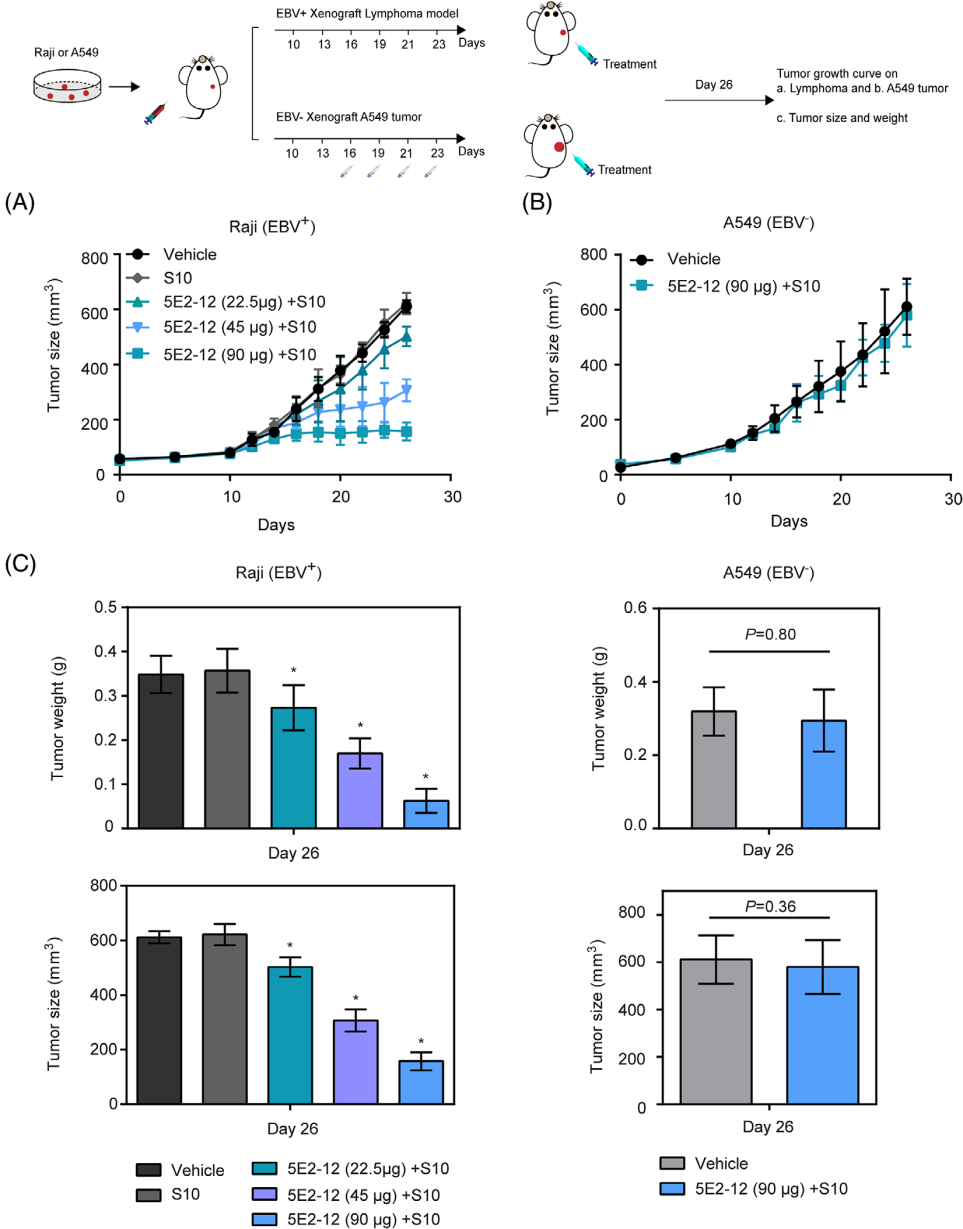
Effect of 5E2‐12 mAb on EBV‐positive lymphoma tumor and EBV‐negative A549 tumor xenografts in mice. The antitumor efficacy of 5E2‐12 mAb was analyzed by measuring the tumor volume during treatment in male BALB/c nude mice carrying: (A) Lymphoma tumor xenografts. *n* = 5 per group. (B) A549 tumor xenografts. *n* = 3 per group. (C) Tumor size and weight were obtained on day 26. *p < 0.05 compared with the vehicle group.

To evaluate the therapeutic efficacy of 5E2‐12 mAb in vivo, we conducted xenograft studies using two EBV‐positive cell line models: the NPC cell line C666‐1 and the Burkitt's lymphoma cell line Raji. In the Raji lymphoma model (Figure 6A), different doses of 5E2‐12 mAb treatment demonstrated significant tumor growth inhibition (TGI) of 17.8, 49.8, and 74.3% compared with the vehicle group (Figures 6A,C and S4A). Importantly, in the EBV‐negative A549 lung cancer xenograft model, there was no inhibition of tumor growth upon 5E2‐12 mAb treatment, indicating the selectivity of the antibody for specifically targeting EBV‐positive tumors (Figures 6B,C and S4B).

**FIGURE 7:**
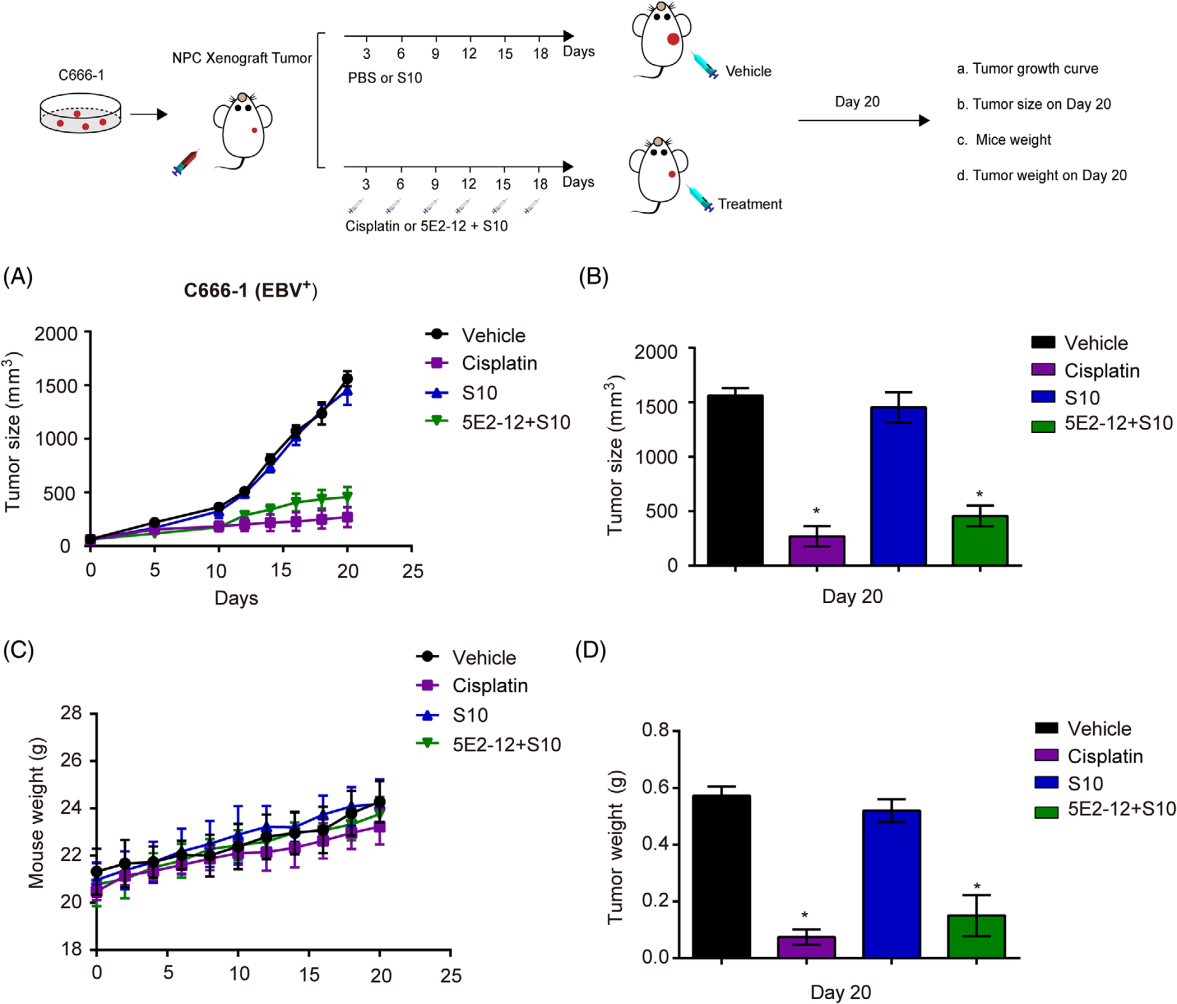
Effect of 5E2‐12 mAb on EBV‐positive NPC tumor xenografts in mice. (A) The antitumor efficacy of 5E2‐12 mAb was evaluated by measuring the tumor volume during treatment in male BALB/c nude mice carrying NPC tumor xenografts. (B) Tumor size was measured on day 20. (C) Mice weight was measured every 2 days. (D) Tumor weight was measured on day 20. Cisplatin, a clinical drug against NPC, as positive control group. *p < 0.05 compared with the vehicle group. *n* = 3 per group.

NPC is commonly treated with a combination of radiotherapy and cisplatin in clinical settings.^26^ In the C666‐1 NPC xenograft model (Figure 7A), we observed significant TGI with both 5E2‐12 mAb and the standard‐of‐care chemotherapeutic agent cisplatin, compared with the vehicle group. The TGI was 70.7% with 5E2‐12 mAb and 82.78% with cisplatin, respectively, indicating the effectiveness of both treatments in suppressing tumor growth (Figures 7B,D and S4C). Furthermore, both 5E2‐12 mAb and cisplatin intratumoral therapy showed no effect on the weight growth of mice (Figure 7C).

These results highlight the therapeutic potential of 5E2‐12 mAb in both NPC and lymphoma models. In the NPC model, the treatment of 5E2‐12 mAb led to significant TGI compared with the control group. In the lymphoma model, 5E2‐12 mAb also exhibited remarkable efficacy in inhibiting tumor growth. These findings support the further development and evaluation of 5E2‐12 mAb as a potential treatment option for EBV‐associated malignancies, offering a targeted approach to combat these challenging diseases.

The selectivity of 5E2‐12 mAb for EBV‐positive tumors was further demonstrated by the lack of TGI in the EBV‐negative A549 xenograft model. This specificity is crucial for minimizing off‐target effects and maximizing the therapeutic benefit in patients with EBV‐associated malignancies. Furthermore, we attempted different administration routes (intravenous and intraperitoneal) of the 5E2‐12 mAb combined with the CPP S10 in the NPC model. While these routes could inhibit the tumor growth, the effect is weaker than the direct intratumoral injection (Figure S1).

Overall, our in vivo studies provide strong evidence for the therapeutic efficacy of 5E2‐12 mAb in inhibiting tumor growth in both NPC and lymphoma models, with the mAb exhibited potent activity in lymphoma model. These findings support the further development and evaluation of 5E2‐12 mAb as a potential treatment option for EBV‐associated malignancies, offering a targeted approach to combat these challenging diseases.

### 2.7 5E2‐12 mAb targets EBNA1 and reduces the expression of EBV genes in vivo

**FIGURE 8:**
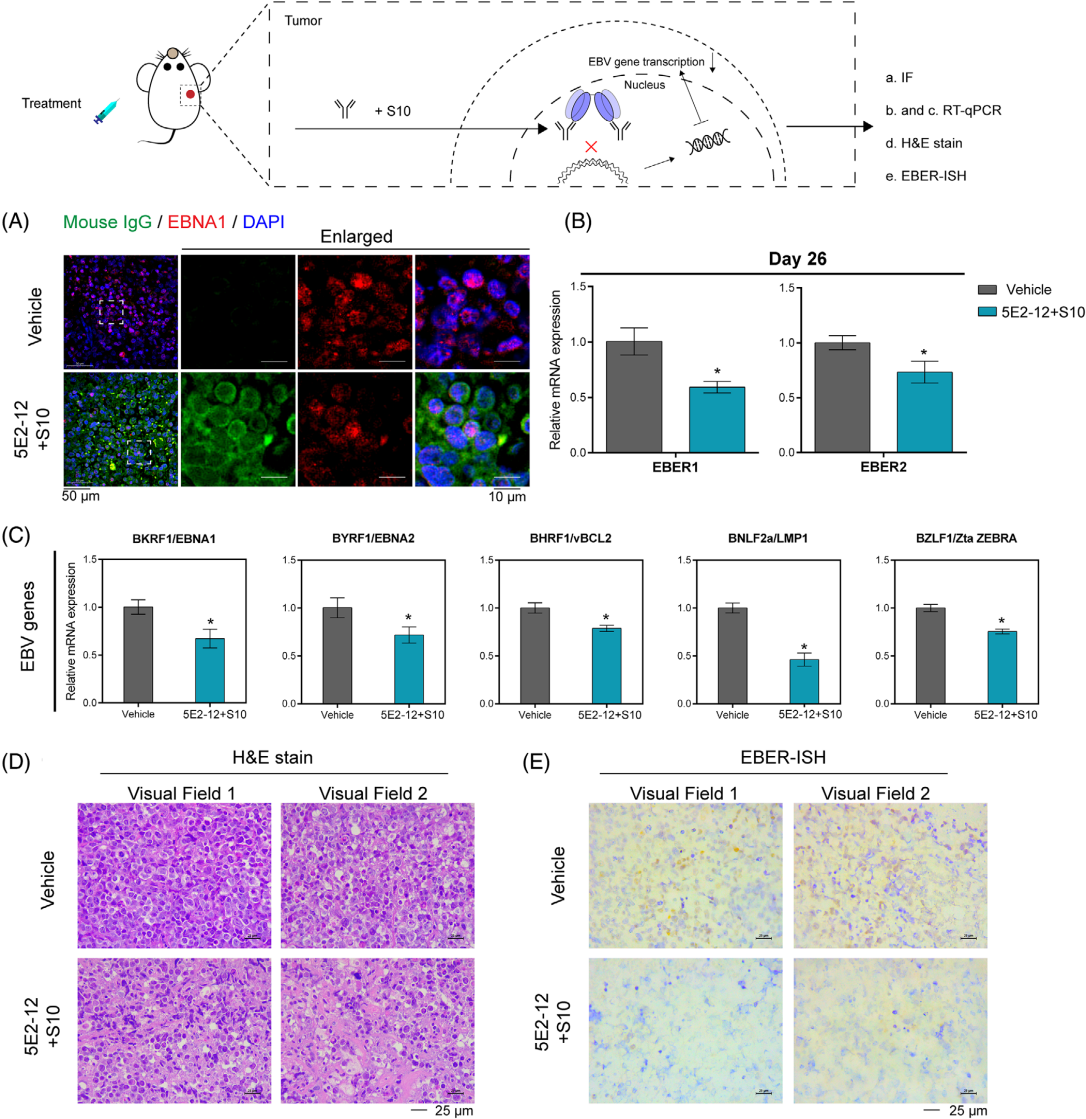
Effect of 5E2‐12 mAb on EBNA1 and EBV genes expression in EBV‐positive NPC tumor xenografts. (A) IF was used to observe the localization of EBNA1 (red) and 5E2‐12 (green) using a confocal microscope in C666‐1 NPC xenografts. Scale bar: 50 µm, enlarged scale bar: 10 µm. RT‐qPCR was employed to measure the expression of: (B) EBV‐encoded RNA (EBER1 and EBER2), and (C) EBV genes, including BKRF1, BYRF1, BHRF1, BNLF2a, and BZLF1. (D) H&E staining was used to detect tumor tissues destroyed by 5E2‐12 + S10. Two random visual fields were placed. Scale bar 25 µm. (E) EBER‐ISH was employed to determine EBER levels in EBV‐positive NPC tumors. Two random visual fields were placed. Scale bar 25 µm. *p < 0.05 compared with the vehicle group. *n* = 3 per group.

To investigate the effect of intracellular 5E2‐12 mAb on EBNA1 and EBV genes, we employed confocal microscopy to examine histopathologic sections of EBV‐positive NPC tumors. Our analysis revealed the colocalization of EBNA1 with the intracellular 5E2‐12 mAb, providing compelling evidence that the antibody specific targets EBNA1 (Figure 8A).

Treatment with 5E2‐12 mAb resulted in a significant reduction in the EBV copy number within tumor tissues, as indicated by the inhibitory effect on the expression of the EBV‐encoded small RNAs (EBER1 and EBER2) detected by reverse transcription quantitative polymerase chain reaction (RT‐qPCR) (Figure 8B). Additionally, our analysis demonstrated that treatment with 5E2‐12 mAb led to a reduction in the expression of various EBV‐encoded genes, including those coding for EBNA1, EBNA2, LMP1, and ZTA (Figure 8C and Table S2). These findings suggest that 5E2‐12 mAb exerts its inhibitory effects on the growth of EBV‐positive tumors by specifically binding to EBNA1, which is closely associated with the loss of the viral genome.

To explore the killing effect of 5E2‐12 mAb on tumors, we performed H&E stain for tumor tissues. Histological analysis of the tumor section from the vehicle control group revealed deeper nuclear staining, abundant cytoplasm, and tight arrangement, indicative of active cell proliferation. In contrast, the tumor sections from the 5E2‐12 + S10 treatment group displayed dispersed cells with decreased density, cell rupture, and cytoplasmic leakage, indicating extensive cell deaths (Figure 8D).

Furthermore, EBER in situ hybridization results showed that 5E2‐12 + S10 treatment significantly reduced the copy number of EBERs in tumor tissues, indicating that EBV latent infections were suppressed (Figure 8E).

The colocalization of EBNA1 and 5E2‐12 mAb observed in the histopathologic sections of NPC tumors provides direct evidence for the specific binding of 5E2‐12 to EBNA1 within the cellular context. Additionally, the inhibitory effect of 5E2‐12 mAb on the EBV copy number indicates a potential interference with viral replication or maintenance in tumor tissues.

The significant reduction in EBV copy number observed upon 5E2‐12 mAb treatment suggests that the antibody may disrupt the stability or replication of the viral genome. The EBERs, noncoding RNAs abundantly expressed in EBV‐positive cells, are commonly used as markers for EBV infection. The inhibitory effect of 5E2‐12 mAb on the EBER signal further supports its ability to interfere with EBV replication or persistence in tumor tissues.

Moreover, the downregulation of various EBV‐encoded genes, including EBNA1, EBNA2, LMP1, and ZTA, upon 5E2‐12 mAb treatment indicates a broader impact on the expression of viral genes critical for EBV‐associated tumor growth and survival. These findings suggest that 5E2‐12 mAb may disrupt key regulatory pathways controlled by EBNA1 and other viral proteins, leading to the inhibition of tumor growth.

In summary, our investigation into the effect of intracellular 5E2‐12 mAb on EBNA1 and EBV genes provides valuable insights into the underlying mechanisms of action. The colocalization of EBNA1 and 5E2‐12 mAb demonstrates the specific targeting of EBNA1 by the antibody within the cellular context. 5E2‐12 mAb treatment significantly reduces the EBV copy number and downregulates the expression of EBV‐encoded genes. These findings suggest that 5E2‐12 mAb interferes with the stability, replication, and expression of the viral genome, ultimately leading to the inhibition of EBV‐positive tumor growth.

## 3 DISCUSSION

The latent infection of EBV plays a pivotal role in the development of EBV‐driven tumors. EBV establishes a state of latency in infected cells, where the virus remains dormant and persists within the host without causing active viral replication. This latent phase is characterized by the expression of a limited set of viral proteins, including EBNA1. During latency, EBNA1 has been found to exhibit an unexpected interaction with a specific DNA sequence known as an EBV‐like palindromic sequence.^13^ This palindromic sequence is situated within the human chromosome 11q23 region. The binding of EBNA1 to this sequence has significant implications for the development of EBV‐associated tumors.

In our study, we aimed to investigate the specific binding of EBNA1 to DNA probes derived from both the EBV genome and the human chromosome. To study the binding of EBNA1 to DNA probes, we used an in vitro fluorescence polarization (FP) method, a widely used technique for analyzing protein–DNA interactions. This method allows us to measure the binding affinity between EBNA1 and the DNA probes accurately. Interestingly, our study revealed that DNA probe 2, which represents the 18‐bp sequence derived from the 11q23 region of the human chromosome, exhibited a significantly higher affinity for EBNA1 compared with the other DNA probes derived from the EBV genome. These results indicate that inhibiting the interaction between EBNA1 and the human chromosome, particularly at the 11q23 region, may hold greater therapeutic value in limiting EBV latent infection and inhibiting the growth of EBV‐positive tumors that have already formed.

The targeting of EBNA1 as a therapeutic approach for treating EBV‐related diseases has gained significant attention since the discovery of its crucial function.^27^, ^28^, ^29^, ^30^, ^31^ In recent years, investigation has increasingly focused on identifying small molecule compounds that selectively bind to specific sites on EBNA1 protein.^16^ However, an alternative and promising approach involves the use of mAbs that target specific epitopes at the interface between EBNA1 and the EBV episomes or the host chromosome. This strategy aims to inactivate EBNA1 and provide a potential treatment option for EBNA1‐related diseases. Previous studies have reported the development of small‐molecule compounds that target Site 2 on the EBNA1 DBD, which was considered a more suitable approach for pharmacological intervention.^16^ In the current study, we identified the 5E2‐12 mAb through an epitope‐directed immune screening approach and demonstrated that it binds to Site 1 (Figure S3), which encompasses an intrinsically disordered region (IDR) of EBNA1. IDRs have often been considered ‘‘undruggable’’ by small‐molecule compounds.^32^ Our findings highlight the importance of the EBNA1 residues 461−471, a critical IDR for the binding of EBNA1 to DNA. This provides insights into the potential of utilizing biomacromolecular agents, such as the 5E2‐12 mAb, to target this otherwise challenging region of EBNA1. Targeting IDRs, which are often involved in protein–protein or protein–nucleic acid interactions, represents a promising strategy for the development of novel therapeutic interventions against EBNA1‐dependent biological processes in EBV‐associated malignancies.

Generating mAbs that specifically target relevant epitopes on a protein of interest poses a significant challenge. In our study, we sought to overcome this challenge by analyzing the crystal structure of EBNA1 bound to its cognate DNA to identify potential pharmacological targets. Through this analysis, we successfully identified three epitopes involved in the EBNA1–DNA interaction. Subsequently, we generated the 5E2‐12 mAb, which specifically targets EBNA1 and demonstrated the ability to block DNA binding both in vivo and in vitro. Moreover, treatment with the 5E2‐12 mAb resulted in a reduction in the volume of EBV‐positive tumors, providing evidence of the therapeutic potential of our approach.

Prior study has investigated the use of peptide‐based probes to regulate the formation of EBNA1 homodimer, which indirectly blocking EBNA1's DNA binding.^28^ While these peptide‐based probes have shown promise, the utilization of mAbs for targeting specific epitopes on EBNA1 opens up new avenues for therapeutic interventions. By directly interfering with the EBNA1–DNA interaction, these mAbs hold the potential to disrupt the crucial functions of EBNA1 in viral latency and tumor development. Additionally, the advantages of antibodies, such as their prolonged activity and high specificity, enhance their potential as therapeutic agents.

While conventional antibody therapies have faced challenges in targeting intracellular proteins that drive diseases, there has been ongoing progress in the development of mAbs that specifically target intracellular targets.^19^, ^33^ To facilitate the intracellular delivery of antibodies, various methods have been investigated, including physical transduction, direct intracellular expression, nanomaterial‐based delivery, and the use of CPPs.^34^, ^35^One approach that has shown promise is the use of clathrin‐mediated endocytosis to transport antibodies targeting intracellular proteins such as Ras.^19^ This method has demonstrated a reduction in tumor xenograft volume in mice. Building on these advances, our study utilized an amphiphilic CPP known as S10. This CPP efficiently delivered the 5E2‐12 mAb into cells or tumor tissues, representing a significant step forward in the field of intracellular antibody delivery. It is important to acknowledge that our current data primarily focuses on the intratumoral administration of the 5E2‐12 mAb. We have not yet explored other administration routes or systemic delivery methods. However, intratumoral administration has its own advantages, particularly in targeting localized tumor sites and minimizing off‐target effects. Further investigation is required to assess the feasibility and efficacy of alternate delivery strategies, such as systemic circulation or targeting specific cell types. The use of CPPs for intracellular antibody delivery holds tremendous potential, but several challenges remain. Achieving efficient cellular uptake, maintaining antibody stability within the intracellular environment, and minimizing potential cytotoxicity are crucial considerations in the development of effective intracellular antibody therapies. Ongoing research efforts aim to address these challenges and optimize the delivery and therapeutic efficacy of intracellular antibodies.

Xenograft studies, while valuable for preclinical evaluation, often have limitations in terms of clinical diversity and the representation of the complex tumor microenvironment. To address these limitations and enhance the translational relevance of our findings, we incorporated different cell lines in our study to assess the in vivo efficacy of the 5E2‐12 mAb. In our experimental design, we specifically selected two EBV‐positive cell lines that originated from different sources and exhibited distinct cell types, genetic backgrounds, and latent programs. By utilizing these diverse cell lines, we aimed to capture a broader spectrum of the heterogeneity observed in EBV‐positive tumors. Remarkably, despite the variations in origin and genetic characteristics, both cell lines exhibited similar responses to the treatment with 5E2‐12 mAb. This consistency in response reinforces the potential therapeutic value of the 5E2‐12 mAb in targeting intracellular proteins in EBV‐positive tumors. Furthermore, we included an EBV‐negative cell line as a control in our study. Notably, this cell line demonstrated an opposite response to the 5E2‐12 treatment, further emphasizing the specificity of the antibody toward EBNA1 and its relevance in the context of EBV‐associated diseases.

Although clinical research in this area is limited, our study provides a valuable foundation for the identification and evaluation of mAb targeting intracellular proteins, particularly in the context of early‐stage EBV‐positive tumors. The consistent responses observed across different EBV‐positive cell lines suggest that the 5E2‐12 mAb holds promise as a potential therapy option for the treatment of such tumors.

## 4 MATERIALS AND METHODS

### 4.1 Protein expression and purification

The protein expression and purification process followed the methods described in a previous study.^36^Residues 461–607 of the EBNA1 DBD from the B95.8 strain (GenBank accession: NC_007605.1) were cloned into the pET28a vector containing the small ubiquitin‐like modifier 3 (SUMO) gene. The N‐terminus of the EBNA1 DBD was fused with a 6 × histidine (6 × His) tag and a SUMO protein tag. The fusion protein was expressed in *E. coli* BL21(DE3) cells and purified using Ni‐NTA resin (L00250; Genscript). After an overnight digestion with ULP1 protease, the protein was further purified using Ni‐NTA resin and a size exclusion column (Superdex 75 Increase 10/300; Cytiva). The purified proteins were collected and concentrated to a concentration of 10−20 mg/mL in a buffer containing 20 mM Tris (pH 7.5) and 0.5 M NaCl. They were then stored at −80°C.

The EBNG1, EBNG2, and EBNG3 proteins were expressed in 293F cells followed the previous methods^37^ and purified using Protein A resin (L00464; Genscript). The 5E2‐12 mAb was purified using Protein G resin (L00209; Genscript).

The EBNA1 DBD mutant 1 (mut1) and mutant 2 (mut2) fused with a 6 × His tag and a SUMO protein tag were expressed in *E. coli* BL21(DE3) cells and purified using Ni‐NTA resin. In mut1, we mutate 461−471 residues in EBNA1 DBD, while in mut2, these residues were removed. The purity of all proteins was assessed by Coomassie brilliant blue R250 staining after SDS‐PAGE.

### 4.2 Mouse immunization

To immunize the mice, 6–8 weeks old female BALB/c mice were used. The immunogens, either EBNA1–DBD or EBNG1, EBNG2, EBNG3, were emulsified with an adjuvant. The immunization was performed at 2‐week intervals. Multiple point injections of the immunogens were administered on the back of the mice.

For the primary immunization, 100 µg of EBNA1–DBD or 50 µg EBNG1, EBNG2, and EBNG3 proteins were injected. In the subsequent boost immunizations, 50 µg of EBNG1, EBNG2, and EBNG3 proteins were administered.

Mice serum samples were collected before the immunization as a control, and after the final immunization for further analysis using ELISA assays. The ELISA analyses were performed to assess the antibody response in the serum samples, specifically measuring the binding of the antibodies to the respective immunogens.

### 4.3 Enzyme‐linked immunosorbent assay

To determine the binding specificity and affinity of serum antibodies to EBNA1 DBD, ELISA assays were performed using a 96 well half‐area plate (Corning). The plate was coated with 25 ng of EBNA1 DBD and incubated at 4°C overnight. After coating, the plate was blocked using a 5% slim milk solution. The plate was then washed using PBST (PBS buffer with 0.05% Tween 20) to remove any unbound components.

For the analysis of serum samples, different dilutions of the serum were prepared and added to the respective wells of the plate. The plate was incubated for 1 h at 37°C to allow binding of antibodies present in the serum to the coated EBNA1 DBD.

After incubation, the plate was washed again with PBST to remove any unbound antibodies. The bound antibodies were then detected using a horseradish peroxidase (HRP)‐conjugated goat anti‐mouse IgG antibody (Bioss; bs0296) at a dilution of 1:5000. The plate was incubated with the secondary antibody for a specific period of time.

Following another round of washing with PBST, the bound antibodies were visualized by incubating the plate with BD OptEIA™ TMB Substrate Reagent Set (Biosciences; 555214). The TMB substrate undergoes a color change in the presence of HRP, resulting in a measurable signal.

The absorbance of the developed color was read at 450 nm using a Tecan Spark (M1000PRO) microplate reader. The absorbance value at 450 nm is indicative of the binding of antibodies to the coated EBNA1 DBD.

### 4.4 Hybridomas and mAb preparation

According to the previous methods,^38^ hybridomas were generated by fusing the spleen cells of immunized mice, which displayed high serum antibody titers, with SP2/0 myeloma cells. mAbs were produced through in vivo antibody secretion in mouse ascites. Briefly, monoclonal hybridomas were isolated from multiple 96‐well plates. Approximately 5 × 10^5^ monoclonal hybridomas cells were then injected intraperitoneally into 8‐week‐old male BALB/c mice that had been pretreated with Freund's incomplete adjuvant. After 10 days, the mice were euthanized, and the ascites fluid containing the secreted mAbs were collected. The antibodies were purified using the affinity chromatography method described above and stored in sterile phosphate buffered saline (PBS).

### 4.5 Surface plasmon resonance

The EBNA1 DBD was immobilized as a stationary phase onto activated 3D Dextran sensor chips. The coupling of EBNA1 DBD onto the sensor chips was carried out overnight at 4°C. After the coupling reaction, the sensor chips were blocked using a 1 M ethanolamine aqueous solution for 30 m. Following this, the sensor chips were dried using nitrogen gas and stored at 4°C for later use.

To measure the affinity constant (*K*_D_), different concentrations of the 5E2‐12 mAb were used as the flow phase. The 5E2‐12 mAb was loaded onto a PlexArray HT A100 instruments at a flow rate of 2 µL/s. The sensor chip captured the binding interactions between the immobilized EBNA1 DBD and the 5E2‐12 mAb.

The obtained sensor data, representing the binding responses, were then analyzed using BIAevaluation Software to calculate the affinity constant (*K*_D_), which is a measure of the binding affinity between the 5E2‐12 mAb and the EBNA1 DBD.

### 4.6 Fluorescence polarization

A DNA probe labeled with carboxyfluorescein (FAM) was synthesized by Tsingke Biotech Co., Ltd. The probe sequence is provided as follows:

DNA 01: FAM—GGGTAGCATATGCTACCC (5′ to 3′)
DNA 02: FAM—GGGTAACCACTGTTACCC (5′ to 3′)

To detect the DNA‐binding capacity of EBNA1 DBD, a 96‐well plate was prepared. 50 µL of EBNA1 DBD at different concentrations ranging from 20 to 0.31 µM was added to 50 µL of a 20 nM solution of the 5′‐FAM DNA probe. The binding reaction was performed in a buffer containing 200 mM NaCl, 20 mM Tris–HCl pH 7.5, 1 mM MgCl_2_, and 0.01% CHAPS.

After incubating the mixture for 20 m, the FP signals were measured using a Tecan Spark microplate reader. The excitation wavelength was set at 485 nm, and the emission wavelength was set at 535 nm. The obtained FP signals were used to determine the effective concentration (EC_50_) value, which represents the concentration of EBNA1 DBD required to bind to half of the DNA probe.

For the competitive assay of the 5E2‐12 mAb or compound, a similar setup was followed. 10 µL of the 5E2‐12 mAb or SC7 (positive control) at different concentrations (ranging from 6.6 µM to 0.0066 nM or 0.2 M to 0.2 µM) or PBS (negative control) was added to 45 µL of a 10 µM solution of EBNA1 DBD. After a 20 m incubation, 45 µL of a 20 nM solution of the 5′‐FAM DNA probe was added to each well and incubated for an additional 20 m. The FP signals were immediately measured.

The obtained data from the competitive assay were used to calculate the inhibitory concentration (IC_50_) value, which represents the concentration of the 5E2‐12 mAb or compound required to inhibit the binding of EBNA1 DBD to the DNA probe by 50%.

The EC_50_ and IC_50_ values were calculated using GraphPad Prism 6.0 software, which is commonly used for data analysis and curve fitting in scientific research.

### 4.7 Cell lines

The following cell lines were used in this study: C666‐1, HK1, Raji, CNE‐2, and A549. C666‐1 and HK1 cells were obtained from iCell Bioscience Inc., Raji cells from Pricella Biotechnology Co., Ltd., CNE‐2 cells from Bohui Biotechnology Co., Ltd., and A549 cells from Fenghui Biotechnology Co., Ltd. All cell lines were maintained in RPMI‐1640 medium supplemented with 10% fetal bovine serum and 1% penicillin–streptomycin solution. Cultures were incubated at 37°C in a humidified atmosphere containing 5% CO_2_.

### 4.8 Cell proliferation and cytotoxicity assay

The Cell Counting Kit (CCK‐8; Yeasen) was utilized to assess cell proliferation in this study. Briefly, a total of 5 × 10^4^ cells per well (100 µL) were seeded into a 96‐well plate. The 5E2‐12 mAb and S10 (with S10 serving as a negative control) were premixed at different concentrations and then added to the plate. After a 24 h incubation period, 10 µL of CCK‐8 solution was added to each well and incubated for 1 h. Subsequently, the absorbance at 450 nm was measured using the Tecan Spark microplate reader. The CC_50_ value was calculated using GraphPad Prism 6.0.

The lactic dehydrogenase (LDH) Cytotoxicity Assay Kit (Beyotime) was used to assess cytotoxicity in this study. Briefly, a total of 5 × 10^4^ cells per well (150 µL) were seeded into a 96‐well plate. The 5E2‐12 mAb and S10 (with S10 serving as a negative control) were premixed at different concentrations and then added to the plate. After a 23 h incubation period, 15 µL LDH release reagents were added into the control group. After centrifugation of 400×*g* for 5 m, 120 µL of the supernatant from each well was taken and added to 60 µL of LDH detection working solution, followed by incubation for 1 h at room temperature. Subsequently, the absorbance at 490 nm was measured using the Tecan Spark microplate reader. The CC_50_ value was calculated using GraphPad Prism 6.0.

### 4.9 DNA synthesis assay

BeyoClick™ EdU‐647 stain was employed to detect DNA synthesis. The experimental procedures varied for the adherent C666‐1 cells and the suspension Raji cells.

For the adherent C666‐1 cells, a total of 1 × 10^6^ cells per well were seeded into a six‐well plate. After a 12‐h incubation period, a mixture of 5E2‐12 mAb and S10 (with S10 at the same concentration as the negative control) was added. After 24 h treatment, a 20 µM EdU solution was introduced to each well and incubated for 2 h. Following the incubation, the cells were washed with PBS and sequentially fixed with 4% paraformaldehyde. They were then incubated with PBS containing 0.3% Triton X‐100. Finally, a click reaction with Azide 647 was catalyzed by Cu^2+^. The resulting EdU signals were observed under a fluorescence microscope at 10× magnification, and the signal images were analyzed using Image J software.

For the suspension Raji cells, a total of 2 × 10^5^ cells per well, along with a mixture of the 5E2‐12 mAb and S10 (with S10 at the same concentration as the negative control), were added to a 48‐well plate. Subsequently, a 20 µM EdU solution was added to each well and incubated for 2 h. The cells then underwent the same fixation, permeability, and click reaction steps as the adherent cells. Finally, the EdU signals were detected using a flow cytometer.

### 4.10 ChIP assay and RT‐qPCR

A total of 5 × 10^6^ Raji cells per flask were cultured in two T25 culture flasks. One flask was treated with a mixture of the 5E2‐12 mAb and S10, while the other was treated with S10 alone. Intracellular proteins were cross‐linked with DNA using 1% formaldehyde, and the crosslinking was stopped with 0.125 M glycine. After resuspending the cells in SDS lysis buffer, DNA binding with EBNA1 was disrupted using ultrasound (30% power, 5 s on, 10 s off, repeated 15 times). A 2 µg mouse anti‐EBNA1 antibody (sc‐81581; Santa Cruz Biotechnology) was used to precipitate EBNA1, and Protein A/G resin was employed to bind the mouse antibody. After washing and elution of the resin, the supernatant was subjected to de‐crosslinking. Finally, qPCR was performed to quantify the levels of two EBV sites (DS‐50 and Qp), one cellular site (PITPNB), and the OriLyt locus, which cannot bind to EBNA1 in vivo. Mouse IgG was used as a negative control.

To quantify viral gene expression in cells and tumor tissues, total RNA was extracted and subjected to RT to generate complementary DNA (cDNA). The primer sequences used for qPCR are provided in Table 1. The quantitation of PCR data was used 2^−ΔΔCT^.

**TABLE 1:** Primers for qPCR and ChIP assay.

Gene/locus	Primer name	Assay	Sequence (5′ to 3′)
QP/EBV	QP F	ChIP	AAATTGGGTGACCACTGAGGGAGT
QP/EBV	QP R	ChIP	ATAGCATGTATTACCCGCCATCCG
DS‐50/EBV	DS‐50 F	ChIP	ATGTAAATAAAACCGTGACAGCTCAT
DS‐50/EBV	DS‐50 R	ChIP	TTACCCAACGGGAAGCATATG
OriLyt/EBV	OriLyt F	ChIP	TCGCCTTCTTTTATCCTCTTTTTG
OriLyt/EBV	OriLyt R	ChIP	CCCAACGGGCTAAAATGACA
PITPNB/Cell	PITPNB F	ChIP	TTGCGGTGACTGGTTTTCTG
PITPNB/Cell	PITPNB R	ChIP	AAGGAGGTGGGCCCTGACT
GAPDH	GAPDH‐F	qPCR	CTCTGCTCCTCCTGTTCGAC
GAPDH	GAPDH‐R	qPCR	AGTTAAAAGCAGCCCTGGTGA
BKRF1	BKRF1‐F	qPCR	GTAGGGGATGCCGATTATTTTG
BKRF1	BKRF1‐R	qPCR	CTCCTTGACCACGATGCTTTC
BYRF1	BYRF1‐F	qPCR	CATCTGCTATGCGAATGCTT
BYRF1	BYRF1‐R	qPCR	ATGTGGCTGGACCAACCTG
BNLF2a	BNLF2a‐F	qPCR	GTATTGGCACAAGATGGAAAGC
BNLF2a	BNLF2a‐R	qPCR	CAACTACCAGGCAGATGAGGC
BZLF1	BZLF1‐F	qPCR	CCCAGTCTCCGACATAACCC
BZLF1	BZLF1‐R	qPCR	CAGGCTGTGGAACACCAATG
BHRF1	BHRF1‐F	qPCR	AGGACACTGTAGTTCTGCGTTATC
BHRF1	BHRF1‐R	qPCR	GCTTGGGTCTCCACGGTG
EBER1	EBER1‐F	qPCR	GAGGTTTTGCTAGGGAGGAGAC
EBER1	EBER1‐R	qPCR	GAAGACGGCAGAAAGCAGAGT
EBER2	EBER2‐F	qPCR	AACGCTCAGTGCGGTGCTA
EBER2	EBER2‐R	qPCR	GCCGAATACCCTTCTCCCA

### 4.11 Confocal immunofluorescence

For C666‐1 cells, a total of 1 × 10^6^ cells were plated in two 35 mm confocal dishes: one for the S10 group and the other for the 5E2‐12 + S10 group. After incubation with PBS contained 0.3% Triton X‐100 and blocking with 1% BSA, the cells were incubated with a rabbit anti‐EBNA1 antibody (1:4000, ab316860; Abcam) overnight at 4°C. Subsequently, the cells were incubated with Alexa Fluor® 488‐labeled goat anti‐mouse IgG and Alexa Fluor® 594‐labeled goat anti‐rabbit IgG (1:250, ZF‐0516; ZSGB‐Bio) for 1 h at room temperature. The fluorescence signals were visualized using a laser confocal scanning microscope (FV3000; Olympus).

For tumor tissues, approximately 5 µm thick tissue sections fixed with 4% paraformaldehyde and embedded in paraffin were subjected to antigen retrieval in a 20 µM Tris–EDTA solution (pH 9.0) after dewaxing. Following the same steps as the cells, including blocking and incubation with primary and fluorescent secondary antibodies, the signals were observed using a laser confocal scanning microscope (LSM 880).

### 4.12 Western blot assay

After 24 h treated with PBS in vehicle group or 5E2‐12 + S10 in treatment group, the Nuclear and Cytoplasmic Protein Extraction Kit was used to extract proteins from Raji cells. A total of 60 µg of protein was separated by sodium dodecyl‐sulfate polyacrylamide gel electrophoresis (SDS‐PAGE) and transferred onto a polyvinylidene fluoride (PVDF) membrane. After blocking with 5% skim milk, the membrane was incubated with primary antibodies overnight at 4°C. Horseradish peroxidase (HRP)‐conjugated secondary antibodies (either goat anti‐mouse or anti‐rabbit IgG) were used to bind to the primary antibodies. The blots were then imaged using a Tanon chemiluminescence instrument following enhanced chemiluminescence detection.

### 4.13 Xenograft tumor models

Briefly, a total of 3 × 10^6^ C666‐1 cells, 3 × 10^6^ Raji cells, or 3 × 10^6^ A549 cells were resuspended in cold PBS mixed with 50% cold Matrigel. The cell mixture was then injected subcutaneously into the flank of each 5‐week‐old male BALB/c nude mouse. The mice were randomly divided into four groups for the NPC model and two groups for the lymphoma and A549 tumor models. Tumor size was measured daily until the diameter reached 7−10 mm (*n* = 3 per group).

In the NPC model, the cisplatin group, which served as the positive control, received intratumoral injections of 4 mg/kg twice a week. The treatment group received intratumoral injections of a mixture containing 20 µM of 5E2‐12 mAb and 40 µM of S10 (30 µL) once every 3 days. This corresponds to a dose of 90 µg of mAb and 5 µg of CPP in PBS. In the lymphoma model, different doses of mAb (90, 45, and 22.5 µg) and a consistent of 5 µg CPP were employed. The vehicle group received intratumoral injections of an equal volume of PBS. For intravenous (i.v.) and intraperitoneal (i.p.) injections, the mice were randomly divided into three groups. The two treatment groups received intravenous or intraperitoneal injections of a mixture containing 20 µM of 5E2‐12 mAb and 40 µM of S10 once every 3 days.

Following the administration of treatments, the mice's weight, and tumor volume (calculated as [(length × width^2^)/2]) were measured every other day. In the NPC model, the mice were sacrificed on day 20 after treatment, while in the lymphoma model and A549 negative control group, mice were sacrificed on day 26.

### 4.14 Epitope analysis of antibody binding

To determine the epitope recognized by the 5E2‐12 mAb, we performed a competitive ELISA. Microtiter plates were coated with 5 µg/mL of the EBNA1 DBD. The 5E2‐12 mAb was preincubated with varying concentrations of different EBNA1 peptide immunogens (EBNG1, EBNG2, and EBNG3) before adding the mixture to the EBNA1 DBD‐coated plates. After a 1‐h incubation at 37°C to allow for antibody binding, the bound antibodies were detected using an HRP‐conjugated goat anti‐mouse IgG antibody. The colorimetric reaction was developed with a TMB substrate, and the absorbance at 450 nm was measured using a Tecan Spark plate reader. EBNA1 DBD mutants 1 and 2 were used as negative controls. The competitive binding curves were generated using GraphPad Prism 6.0 software.

### 4.15 Pathological analysis of tumor tissues

Tumor tissue sections were subjected to hematoxylin and eosin (H&E) staining. Briefly, the tissue sections were sequentially soaked in H&E dyes, followed by washing. The stained sections were then examined under a biological microscope, and the results were recorded.

Additionally, EBV‐encoded small RNA (EBER) in situ hybridization (ISH) was performed on paraffin‐embedded tumor tissue sections. Oligonucleotide probes labeled with digoxin and complementary to the EBER transcripts were used for the hybridization (Servicebio, Wuhan, China).

### 4.16 Statistical analysis

Statistical analyses, including determination of EC_50_/IC_50_ values and generation of column graphs, were conducted using GraphPad Prism 6.0 software. The data in this research are presented as mean ± standard deviation.

For comparisons between two groups of data, *p* values were calculated using Student's *t*‐test. In cases where multiple groups were involved, a one‐way analysis of variance was performed for three experimental or biological replicates. A significance level of *p* < 0.05 was considered statistically significant.

## AUTHOR CONTRIBUTIONS

Yongyue Han cloned, expressed, and purified the proteins, identified the antibodies, conducted cell assays and mice models, and wrote the draft. Fang Wu and Ying Zhang studied the mechanism, analyzed the data, and edited the draft. Yuzhe Wu, Yuzhe Wu, and Yuecheng Wang assisted with the in vitro assays, while Xiwen Jiang and Xin Chen provided the materials. Wei Xu supervised this project and the writing process. All authors have read and approved the final manuscript.

## CONFLICT OF INTEREST STATEMENT

The authors declare no conflict of interest.

## ETHICS STATEMENT

The study for BALB/c mice immunization was approved by the Institutional Animal Care and Use Committee (IACUC) of Southern Medical University, approval number: SMUL2022164. The study for BALB/c nude mice of xenograft tumor models was approved by the Institutional Animal Care and Use Committee (IACUC) of Southern Medical University, approval number: SMUL202402018.

## Supporting information



Supporting Information

## Data Availability

The data that support the findings of this study are available from the corresponding author upon reasonable request.
