# Supplementary material for: Structure‐based design of antibodies targeting the EBNA1 DNA‐binding domain to block Epstein–Barr virus latent infection and tumor growth
Source: MedComm (2020). 2024 Oct 10;5(10):e739. doi: 10.1002/mco2.739 (PMC11467371; doi:10.1002/mco2.739)
Supplement: Supplementary file 1 — Supporting Information [file MCO2-5-e739-s001.docx]

**Supplementary materials**

Structure-Based Design of Antibodies Targeting the EBNA1 DNA Binding Domain to Block Epstein-Barr Virus Latent Infection and Tumor Growth

Yongyue Han^1#^, Fang Wu^2#^, Ying Zhang^1#^, Jun Liu^1^, Yuzhe Wu^1^, Yuecheng Wang^1^, Xiwen Jiang^4^, Xin Chen^3^, Wei Xu^1,5*^

^1^Guangdong Provincial Key Laboratory of New Drug Screening & NMPA Key Laboratory for Research and Evaluation of Drug Metabolism & Guangdong-Hong Kong-Macao Joint Laboratory for New Drug Screening, School of Pharmaceutical Sciences, Southern Medical University, Guangzhou, 510515, China.

^2^Affiliated Foshan Maternity & Child Healthcare Hospital, Southern Medical University, Foshan, 528000, China.

^3^Department of Pulmonary and Critical Care Medicine, Zhujiang Hospital, Southern Medical University, Guangzhou, 510280, China.

^4^School of Life Sciences and Biopharmaceuticals, Guangdong Pharmaceutical University, Guangzhou, 510006, China.

^5^Key Laboratory of Infectious Diseases Research in South China, Ministry of Education, Southern Medical University, Guangzhou, 510515, China

#These authors contributed equally

*Correspondences: e-mail addresses: xuwei3322@smu.edu.cn (W.X)

Running title: Epitope-directed EBNA1-mAb curb EBV^+^ tumors


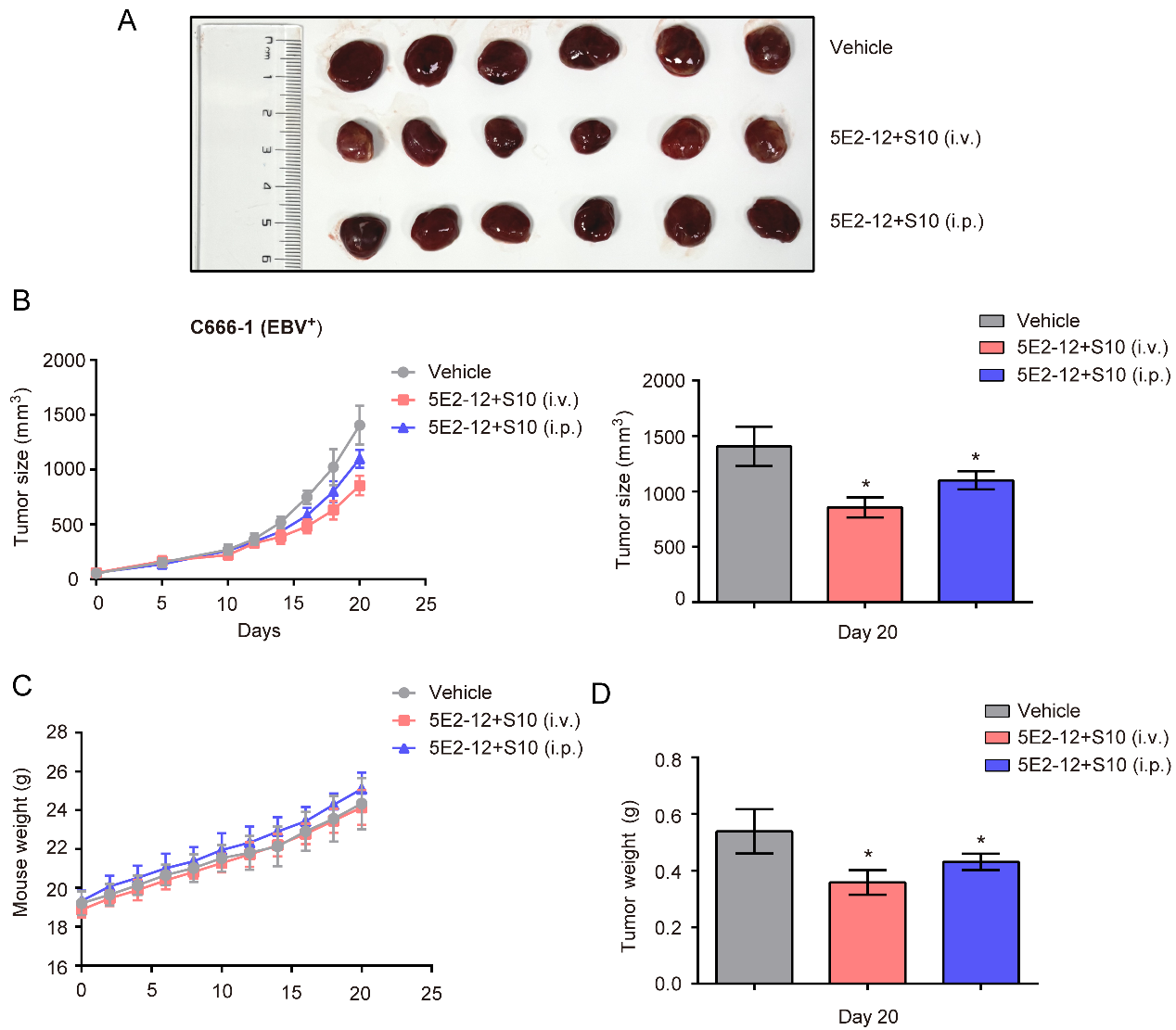


**Figure S1. Anti-Tumor Effects of the 5E2-12 mAb Administered by Intravenous or Intraperitoneal Injection in EBV-Positive NPC Tumor Xenografts in Mice.**

(A) Representative images of the tumors obtained on day 20 of the treatment.

(B) The anti-tumor efficacy of the 5E2-12 mAb administered by intravenous or intraperitoneal injection was evaluated by measuring the tumor volume during the treatment period in male BALB/c nude mice bearing NPC tumor xenografts. The tumor growth curve is shown on the left, and the tumor size on day 20 is shown on the right.

(C) The body weight of the mice was measured every two days during the treatment.

(D) The tumor weight was measured on day 20.

i.v., Intravenous injection; i.p., Intraperitoneal injection. **P* < 0.05 compared to the Vehicle group. n=6 per group.

**
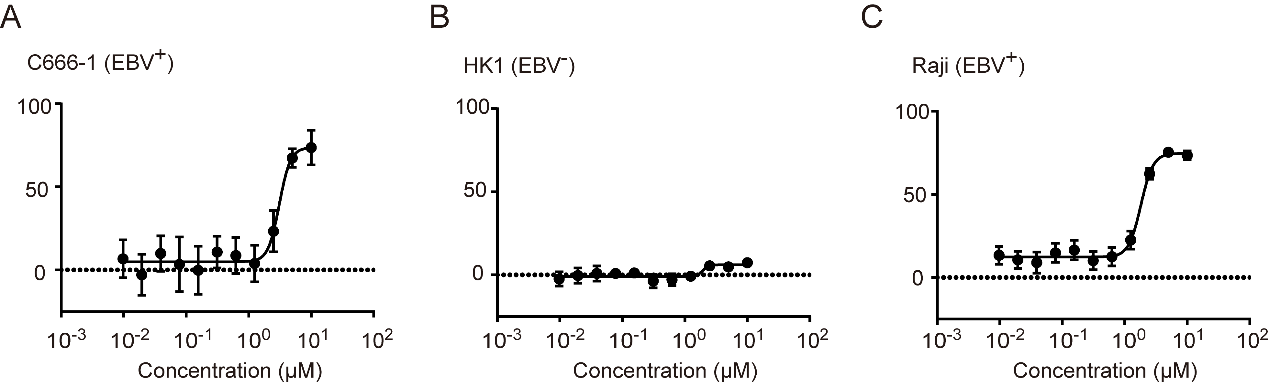
**

**Figure S2. Cytotoxicity Assay of 5E2-12 mAb on C666-1, HK1 and Raji Cells.**

Lactate Dehydrogenase (LDH) assay was utilized to evaluate the cytotoxic effect of 5E2-12 mAb on (A) C666-1 cells: CC_50_=3.09 μM, (B) HK1 cells and (C) Raji cells: CC_50_=1.82 μM.


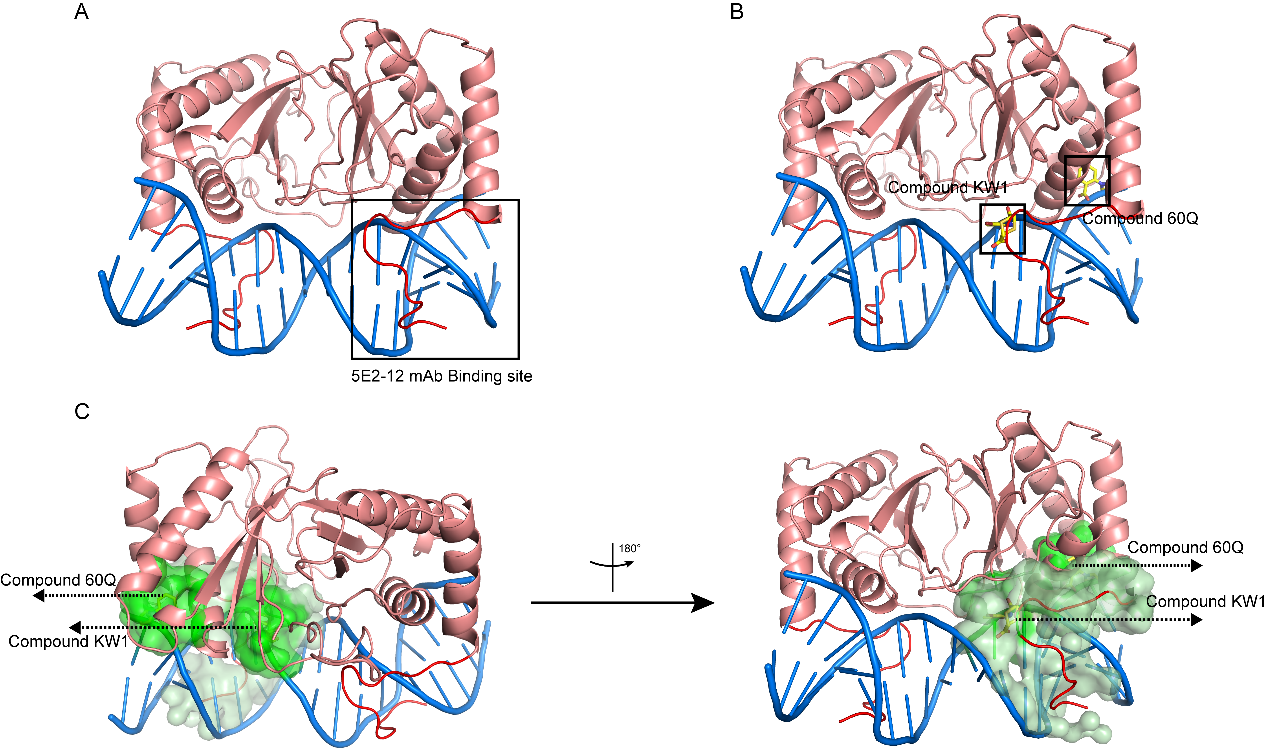


**Figure S3. Comparison of Binding Interfaces between Compounds and 5E2-12 mAb.**

(A) The epitope (Site 1) recognized by the 5E2-12 mAb on the binding interface between the EBV homologous DNA and the EBNA1 DBD.

(B) and (C) The binding interface (Site 2) between the small-molecule compounds (Fragments VK) and the EBNA1 DBD.

PDB: 1B3T, 6NPI.


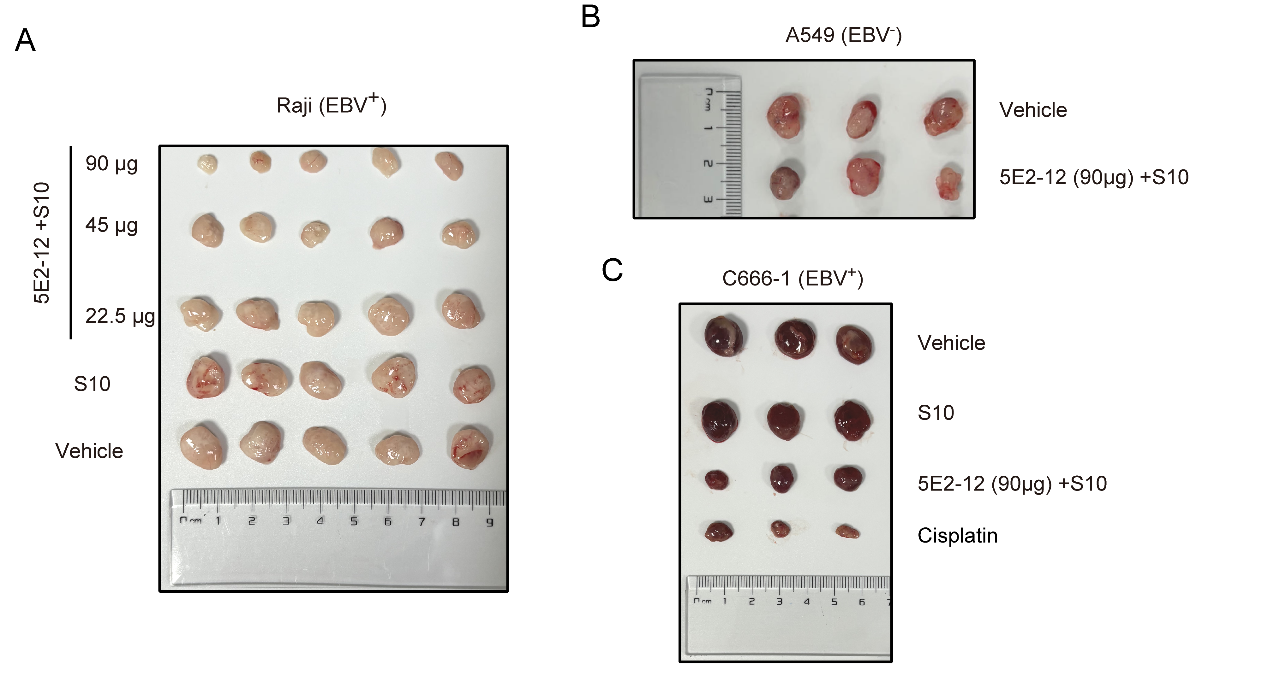


**Figure S4. Comparative Anti-Tumor Effects of the 5E2-12 mAb in EBV-Positive NPC, Lymphoma Tumor Xenografts and EBV-Negative A549 Tumor Xenografts in Mice.**

(A) Representative images of the lymphoma tumors obtained on day 26 of the treatment. n=5 per group.

(B) Representative images of the A549 (EBV-negative) tumor xenografts obtained on day 26. n=3 per group.

(C) Representative images of the NPC tumor xenografts obtained on day 20. n=3 per group.

Table S1. The raw data for RT-qPCR in Figure 4C.

| **S10** | | **5E2-12+S10** | | **S10** | | **5E2-12+S10** | |
| --- | --- | --- | --- | --- | --- | --- | --- |
| Target | Cт | Target | Cт | Target | Cт | Target | Cт |
| GAPDH | 27.211502 | GAPDH | 21.887486 | EBER1 | 17.48455 | EBER1 | 14.969254 |
|  | 27.447281 |  | 21.956726 |  | 18.163944 |  | 14.895919 |
|  | 27.415417 |  | 21.916544 |  | 16.92625 |  | 14.682034 |
| BKRF1 | 23.346453 | BKRF1 | 18.25668 | EBER2 | 22.859255 | EBER2 | 19.750895 |
|  | 23.29915 |  | 18.474352 |  | 23.122505 |  | 19.519651 |
|  | 22.817932 |  | 18.554924 |  | 22.556274 |  | 19.593637 |
| BYRF1 | 26.780609 | BYRF1 | 24.799696 | BZLF1 | 24.057489 | BZLF1 | 19.508446 |
|  | 26.795006 |  | 25.312429 |  | 24.615055 |  | 19.476774 |
|  | 26.557522 |  | 24.600918 |  | 24.61405 |  | 19.538538 |

Table S2. The raw data for RT-qPCR in Figure 8B and 8C.

| **Vehicle** | | **5E2-12+S10** | | **Vehicle** | | **5E2-12+S10** | |
| --- | --- | --- | --- | --- | --- | --- | --- |
| Target | Cт | Target | Cт | Target | Cт | Target | Cт |
| GAPDH | 21.940081 | GAPDH | 21.951908 | BNLF2a | 27.852463 | BNLF2a | 28.93803 |
|  | 21.986109 |  | 22.056051 |  | 27.767513 |  | 28.779537 |
|  | 21.969509 |  | 21.735744 |  | 27.75247 |  | 28.874964 |
| BKRF1 | 34.217258 | BKRF1 | 34.940632 | BZLF1 | 32.318573 | BZLF1 | 32.729445 |
|  | 34.100037 |  | 34.64328 |  | 32.470863 |  | 32.911964 |
|  | 34.037193 |  | 34.368309 |  | 32.392128 |  | 32.600037 |
| BYRF1 | 30.941765 | BYRF1 | 31.149702 | EBER1 | 17.15136 | EBER1 | 17.939173 |
|  | 30.955725 |  | 31.5653 |  | 17.129452 |  | 17.838356 |
|  | 30.705181 |  | 31.183212 |  | 16.85561 |  | 17.484137 |
| BHRF1 | 28.382496 | BHRF1 | 28.861676 | EBER2 | 23.572374 | EBER2 | 23.900835 |
|  | 28.549967 |  | 28.861085 |  | 23.438122 |  | 23.861023 |
|  | 28.551437 |  | 28.641352 |  | 23.463562 |  | 23.93478 |
